# Exploring collaborative strategies to improve patient safety in healthcare organizations: A qualitative study

**DOI:** 10.1371/journal.pone.0341022

**Published:** 2026-01-13

**Authors:** Mohammad Esmaeil Hejazi, Ghasem Alizadeh-dizaj, Shiva Khoshsirat, Samira Raoofi, Akbar Javan Biparva

**Affiliations:** 1 Tuberculosis and Lung Disease Research Center, Tabriz University of Medical Sciences, Tabriz, Iran; 2 Department of Health Information Technology, School of Management and Medical Informatics, Tabriz University of Medical Sciences, Tabriz, Iran; 3 Research Center for Evidence-Based Health Management, Maragheh University of Medical Sciences, Maragheh, Iran; University of Verona, ITALY

## Abstract

**Background:**

Patient safety remains a critical concern in healthcare, necessitating the exploration of collaborative approaches to enhance care quality and outcomes. This qualitative study delves into the significance of interprofessional collaboration, leadership support, patient engagement, and safety culture in driving patient safety initiatives within healthcare settings.

**Method:**

This qualitative phenomenological approach, involving in-depth interviews and document analysis, was conducted to explore the perspectives of healthcare professionals, administrators, patients, policymakers, and researchers involved in patient safety initiatives. Thematic analysis was employed to identify and organize recurring patterns in the data, while interpretive phenomenological analysis was utilized to gain a deeper understanding of participants’ lived experiences. This dual approach revealed key themes related to teamwork, communication, leadership, patient engagement, and best practices in patient safety.

**Results:**

The study revealed that interprofessional collaboration is widely recognized as vital for patient safety, with participants highlighting the importance of effective communication, shared decision-making, and mutual respect among healthcare professionals. Leadership support, organizational structures, and a culture of safety emerged as key enabling factors. Despite the benefits, challenges such as hierarchy, siloed communication, resource constraints, and resistance to change remain significant barriers. Strategies including regular interdisciplinary training, patient engagement initiatives, leveraging technology, and continuous quality improvement processes were identified as effective ways to strengthen collaborative practices.

**Conclusion:**

Promoting interprofessional collaboration, strong leadership, and a robust safety culture are essential for improving patient safety outcomes. Overcoming systemic and interpersonal barriers requires targeted interventions at both individual and organizational levels, with ongoing education, patient participation, and data-driven feedback playing crucial roles in sustaining collaborative efforts. These insights support the adoption of integrated approaches to foster safer and higher-quality care across healthcare settings.

## Introduction

Patient safety, defined by the World Health Organization as “the absence of preventable harm to a patient during the process of healthcare and the reduction of risk of unnecessary harm”, is a cornerstone of high-quality healthcare delivery, representing a fundamental ethical obligation to protect patients from harm and ensure their well-being throughout the care continuum [1–3]. Despite significant advancements in medical science and technology, preventable medical errors and adverse events continue to pose a threat to patient safety, [4–6]. As such, the pursuit of effective **patient safety initiatives** has become a critical imperative for healthcare organizations globally, prompting a reexamination of existing approaches and the exploration of innovative solutions [7,8]. In this context, the concept of collaborative approaches to enhancing **patient safety initiatives** has emerged as a promising paradigm for advancing the quality and safety of care [9,10]. Collaboration among healthcare professionals, administrators, patients, policymakers, and researchers offers a multifaceted and interdisciplinary framework for addressing the complex and multifactorial nature of patient safety challenges [11,12]. By fostering a culture of shared responsibility, transparency, and communication, collaborative initiatives can facilitate the identification of potential risks, the implementation of evidence-based interventions, and the continuous monitoring and improvement of **patient safety initiatives** [13,14].

The significance of collaboration in enhancing **patient safety initiatives** is underscored by the interconnected nature of healthcare systems, which necessitate coordinated efforts across various stakeholders to achieve meaningful and sustained improvements [15]. By engaging in collaborative partnerships, healthcare organizations can leverage diverse perspectives, skills, and resources to develop comprehensive safety strategies that address the root causes of patient harm and promote a culture of continuous learning and improvement [16,17]. Moreover, collaboration can enhance the dissemination of best practices, facilitate the adoption of innovative technologies, and empower patients to play an active role in their own care, thereby promoting a more patient-centric and safety-conscious healthcare environment [10,11,17].

This study brings a fresh perspective by adopting a comprehensive phenomenological approach that explores the lived experiences, perceptions, and practices of diverse stakeholders involved in **patient safety initiatives**. By delving deeply into the collaborative dynamics and interactions shaping patient safety outcomes, the study sheds new light on the intricacies of teamwork, coordination, and communication in healthcare settings. Furthermore, the study distinguishes itself by incorporating perspectives from a wide range of participants, including healthcare professionals, administrators, patients, policymakers, and researchers. This multi-stakeholder approach offers a holistic view of collaborative **patient safety initiatives**, capturing a diverse array of insights and reflections that enrich the analysis and recommendations put forth..

While past studies often focused on measuring outcomes of specific interventions or examining the impact of particular tools and programs [3,11,18,19], they lacked a deep understanding of the lived experiences of those involved in collaborative efforts. Additionally, these studies often focused primarily on healthcare professionals, neglecting the valuable perspectives of patients, administrators, policymakers, and researchers. This study fills this gap by adopting a phenomenological approach, delving into the lived experiences, perceptions, and practices of diverse stakeholders involved in patient safety initiatives.

Despite the acknowledged significance of collaboration in patient safety initiatives, there remains a noticeable gap in the literature regarding how different stakeholders experience and perceive these collaborative efforts. Much of the existing research tends to homogenize the experiences of healthcare teams, overlooking the unique perspectives that patients and various healthcare roles bring to the table. This gap limits not only our understanding of the collaborative dynamics at play but also the efficacy of the patient safety initiatives designed to mitigate risks and errors. Effective collaboration requires an awareness of the distinct roles, expectations, and challenges faced by each stakeholder group, yet such nuances are often underexplored.

This study highlights the perspectives of diverse stakeholders in patient safety initiatives, revealing key themes such as teamwork, patient engagement, communication breakdowns, role clarity, and leadership support, and offers actionable recommendations to strengthen collaboration and contribute to a deeper understanding of best practices and policy development in patient safety.

## Materials and methods

### Research design

This study examined collaborative approaches in patient safety initiatives using a phenomenological perspective to capture the lived experiences, perceptions, and practices of healthcare professionals, administrators, patients, and policymakers. The aim was to understand how collaboration affects patient safety outcomes across various healthcare settings. Phenomenology offered a comprehensive framework to analyze interactions, decision-making processes, and communication patterns that influence the success of collaborative efforts.

### Data collection

Data were collected between July 22, 2024, and August 10, 2024, from Tabriz University of Medical Sciences. A purposive sampling strategy was used to recruit participants representing diverse stakeholder roles involved in patient safety initiatives. The inclusion criteria included at least 2 years of experience in healthcare or policy roles related to patient safety, direct involvement in collaborative patient safety initiatives, and willingness to share their experiences. Exclusion criteria included individuals without relevant experience, not involved in collaborative initiatives, and unwilling to participate.

### Interviews

Semi-structured interviews were conducted both in person and via telecommunication platforms to ensure flexibility and representation. The interview guide was developed based on literature review, established theories, and expert consultation. Sample questions included:

“Can you share your experiences and perspectives on collaborative patient safety initiatives within healthcare settings?”

“Can you elaborate on a specific experience or project related to collaborative patient safety that you found particularly impactful?”

Each interview lasted approximately 60 minutes, allowing sufficient time for in-depth exploration of participants’ experiences and perspectives.

### Document analysis

In addition to interviews, the study incorporated an analysis of relevant literature, reports, and case studies on collaborative patient safety models. This provided a broader contextual understanding of success factors, barriers, and the evolution of collaborative practices.

### Participant overview

The demographic characteristics of the participants are summarized in Table 1, which provides a detailed overview of their roles, gender, age, education, job title/role, years of experience, and geographical locations to illustrate the diversity of perspectives captured in the study. Although the number of patients and policymakers was smaller due to population size and availability, their inclusion was essential to capture multi-stakeholder perspectives.

**Table 1:** The demographic characteristics of the participants.

Participant Role	Number	Gender	Age	Education	Job Title/Role	Years of Experience	Geographic Location
Healthcare Professionals	10	6M/4F	28–45	MD, RN, MSc	Physician, Nurse, Quality Officer	5–20	Urban, Suburban
Administrators	5	3M/2F	32–50	MSc, MBA	Department Head, Program Manager	8–15	Urban
Patients	3	1M/2F	45–70	High School, University	Patient with prior hospital experience	N/A	Rural
Policymakers	2	1M/1F	40–55	MSc, PhD	State Health Officer, Policy Advisor	10	Statewide

### Data analysis

Thematic analysis was employed as outlined by Braun and Clarke (2006). MAXQDA software was used to organize, manage, and code qualitative data systematically. This systematic approach enabled the identification, analysis, and reporting of recurring patterns and overarching themes derived from qualitative data. The analysis was collaboratively conducted by all authors to enhance reliability and mitigate researcher bias.

Thematic analysis involved several steps and shown in Table 2.

**Table 2:** The stages of thematic analysis.

Themes	Categories	Condensed Meaning Units
Factors Affecting Teamwork	Barriers to Collaboration	“Communication breakdowns”
Importance of Patient-Centered Care	Patient Engagement	“Patient involvement is key”
Role of Leadership in Safety	Leadership & Culture	“Leadership support is crucial”

- Familiarization: Researchers read and reread transcripts to immerse themselves in the data.
- Coding: Initial codes were generated from significant statements and phrases reflecting participants’ experiences and perspectives. Codes were organized into categories to facilitate the emergence of overarching themes.
- Theme Development: Codes were grouped into broader themes that represented the main concepts related to collaboration and patient safety.
- Member Checking: Participants were invited to review preliminary findings and provide feedback to enhance validity and ensure accurate representation of their experiences.

#### Interpretive phenomenological analysis (IPA).

IPA was applied to examine the nuanced meanings, perspectives, and emotions embedded within participants’ narratives. This approach allowed the study to uncover deeper layers of understanding regarding collaboration and patient safety. The process involved transforming narratives into meaning units and synthesizing them into fundamental themes while setting aside researchers’ preconceptions.

#### Integration of methods.

Thematic analysis provided a structured overview of recurring patterns, while IPA offered a deeper interpretive understanding. Findings from both methods were integrated to present a comprehensive analysis of collaborative patient safety initiatives.

#### Researcher bias.

Potential bias was acknowledged as a key consideration. To minimize bias, member checking, reflexive discussions, and collaborative coding were conducted. Researchers remained aware of their assumptions throughout the analysis process.

### Ethical considerations

This study was approved by the Research Ethics Committee of Tabriz University of Medical Sciences (Approval Code: IR.TBZMED.REC.1402.904).

Informed written consent: Prior to the commencement of interviews, participants were provided with detailed information about the study objectives, procedures, and privacy measures. Informed written consent was obtained from each participant to ensure transparency and ethical conduct.

Confidentiality: Confidentiality protocols were strictly followed to safeguard the anonymity and privacy of the participants. Data were anonymized, securely stored, and accessible only to the research team.

Voluntary participation: Participation in the study was voluntary, and participants retained the right to withdraw at any stage of the research process without any repercussions.

### Trustworthiness

Member Checking: Participants were invited to review and confirm the accuracy of their contributions during member checking sessions. Feedback was incorporated to validate the findings and enhance credibility.

Peer Debriefing: Regular discussions and debriefing sessions among the research team critically analyzed the data, challenged assumptions, and ensured consistency and reliability of interpretations.

Reflexivity: Researchers maintained reflexivity throughout the study, acknowledging potential biases, assumptions, and personal experiences that could influence data collection, analysis, and interpretation. Reflexivity ensured transparency and rigor in the research process.

### Limitations

The study faced limitations regarding sample size, participant availability, and geographical constraints, as well as potential researcher bias. Efforts to mitigate these limitations included detailed reporting, methodological transparency, purposive sampling, and reflexive analysis.

## Results

### Importance of interprofessional collaboration

Participants consistently emphasized the critical importance of interprofessional collaboration in promoting patient safety initiatives within healthcare settings. The synergistic efforts of diverse healthcare professionals working together were seen as key in preventing medical errors, improving care coordination, and enhancing patient outcomes. Effective communication, shared decision-making, and mutual respect emerged as essential components.

Participants highlighted the need for regular interdisciplinary meetings, team-based care planning, and role clarity. The participants stated:

“I consider effective communication, mutual respect among team members, and shared decision-making processes as crucial for successful interprofessional collaboration in promoting patient safety.” (Participant 3, Healthcare Professional)

“Recognizing and respecting the unique contributions of each team member is crucial for successful interprofessional collaboration.” (Participant 8, Healthcare Professional)

### Leadership and organizational support

Leadership commitment, clear accountability structures, and supportive organizational policies were identified as crucial enablers. Participants emphasized leadership visibility, role modeling, and promoting a culture of psychological safety. Organizational initiatives such as quality improvement projects and regular feedback mechanisms were highlighted. The participants stated:

“Strong leadership support and a culture that values and prioritizes patient safety are key drivers for successful collaborative initiatives.” (Participant 7, Administrator)

“Without top-level endorsement and resources, it can be challenging to implement sustainable changes.” (Participant 12, Healthcare Professional)

### Barriers to collaboration

Despite recognizing the benefits of collaborative practices, participants discussed barriers including hierarchical dynamics, siloed communication structures, turf battles, and competing priorities. Limited resources, time pressures, and resistance to change were also noted. Strategies to overcome these barriers included cultural change, interprofessional training, fostering trust, and addressing system-level factors. Participants mentioned:

“I have faced barriers such as hierarchical dynamics, siloed communication structures, and time pressures that have hindered effective teamwork.” (Participant 5, Healthcare Professional)

“Addressing these barriers requires a nuanced approach considering individual perspectives and motivations.” (Participant 15, Policymaker)

### Patient engagement and safety culture

Participants underscored the importance of involving patients as partners, promoting open communication, and fostering transparency and accountability. Initiatives included patient and family advisory councils, patient-led safety rounds, and patient-reported outcomes. A strong safety culture where all stakeholders feel empowered was identified as essential. Participants stated:

“Patient engagement and a strong safety culture are foundational elements of collaborative patient safety efforts.” (Participant 19, Patient)

“Empowering healthcare professionals to take ownership of safety processes can also enhance patient safety outcomes.” (Participant 6, Healthcare Professional)

### Best practices and lessons learned

Insights included interdisciplinary team training, simulation exercises, and regular debriefing sessions. Technology tools such as electronic health records, decision support systems, and secure messaging platforms were identified as beneficial. Continuous quality monitoring, feedback loops, and data-driven improvement processes were emphasized.

### Participants expressed

“Ongoing interdisciplinary training, simulation exercises, and technology tools have been instrumental in enhancing teamwork and communication skills.” (Participant 4, Healthcare Professional)

“Promoting a culture of continuous learning and improvement involves recognizing individual achievements and providing professional development opportunities.” (Participant 11, Administrator)

The frequency of main and sub-themes among stakeholder groups is shown in Table 3.

**Table 3:** Frequency of major themes and sub-themes across stakeholder groups.

Theme/ Sub-theme	Healthcare Professionals (n = 10)	Administrators (n = 5)	Patients (n = 3)	Policymakers (n = 2)	Description of Sub-themes
1. Interprofessional Collaboration	85%	80%	60%	75%	Communication quality, shared decision-making, respect for roles
Effective Communication	80%	60%	55%	70%	Perceived as essential for reducing errors
Shared Decision-Making	75%	70%	40%	60%	Involvement in team choices
2. Leadership & Organizational Support	78%	90%	40%	88%	Leadership visibility, psychological safety, structural support
Leader Role Modeling	70%	85%	35%	80%	Leadership behaviors influencing safety climate
Supportive Policies	65%	90%	30%	88%	Organizational rules facilitating collaboration
3. Barriers to Collaboration	70%	65%	35%	60%	Hierarchical dynamics, siloed communication, turf battles
Hierarchical Dynamics	68%	50%	20%	45%	**Definition:** Patient safety
Siloed Communication Structures	72%	55%	30%	50%	**Definition:** Fragmented or isolated information flow among departments
Turf Battles	60%	45%	10%	40%	**Definition:** Competition among specialties that obstructs cooperation
4. Patient Engagement	60%	55%	90%	50%	Shared responsibility, patient advisory roles
Transparency & Shared Communication	55%	40%	85%	50%	Open disclosure and information exchange
Patient Participation in Safety Efforts	50%	45%	90%	40%	Inclusion in reporting, safety rounds
5. Safety Culture	82%	88%	75%	70%	Accountability, reporting culture, psychological safety
Learning Culture	78%	85%	60%	65%	Continuous improvement orientation
Reporting & Feedback Systems	80%	90%	70%	75%	Systems supporting detection and correction of errors

## Discussion

The study’s findings underscore the critical importance of collaborative patient safety practices in healthcare settings and offer valuable insights into strategies that can drive improvements in care quality, patient outcomes, and organizational culture. By examining key themes such as interprofessional collaboration, leadership support, patient engagement, safety culture, and best practices in patient safety initiatives, we can gain a deeper understanding of the complex interplay of factors that influence patient safety and quality of care delivery.

Interprofessional collaboration stands out as a foundational element of effective healthcare teamwork, communication, and care coordination. By fostering a collaborative environment where healthcare professionals from different disciplines work together seamlessly, organizations can harness the collective expertise and skills of their teams to optimize care and minimize errors. Interprofessional collaboration not only enhances patient safety but also promotes innovation, professional satisfaction, and a culture of shared responsibility among team members. Leveraging the diverse perspectives and strengths of multidisciplinary teams can lead to more comprehensive, holistic, and patient-centered care delivery [20,21].

Leadership support emerged as a crucial driver of collaborative patient safety efforts, with strong and visionary leadership playing a pivotal role in creating a culture of safety and continuous improvement. Leaders who prioritize patient safety, set clear expectations, allocate resources, and provide guidance and support for quality improvement initiatives can inspire their teams to prioritize collaboration, transparency, and ongoing learning. Effective leadership fosters a culture of open communication, accountability, and shared values, which are essential for promoting patient safety, enhancing care quality, and driving organizational success [22,23].

Barriers to collaboration were identified, including hierarchical structures, communication breakdowns, lack of standardized processes, and time constraints. Addressing these barriers requires a comprehensive approach including education, training, organizational change initiatives, and system-level interventions. By breaking down silos, fostering open communication, and promoting team-based care, healthcare organizations can overcome these challenges and create a culture of collaboration that supports patient safety and quality improvement [24,25].

Patient engagement and safety culture were highlighted as critical components. Engaging patients as partners in their care, promoting shared decision-making, and integrating patient feedback into safety improvement efforts are essential for delivering patient-centered care and building trust. Fostering a safety culture that prioritizes transparency, reporting, and continuous learning is crucial for successful collaborative patient safety initiatives [18,26].

Best practices and lessons learned include interdisciplinary team training, technology utilization, continuous quality monitoring, feedback mechanisms, and data-driven improvement processes. Implementing these strategies and sharing best practices can drive sustainable improvements, enhance care quality, and promote a culture of collaboration and continuous learning [14,27–29].

The study’s findings highlight the importance of fostering a collaborative culture in healthcare, emphasizing interprofessional teamwork, leadership engagement, patient-centered approaches, and effective use of technology. Policymakers and healthcare organizations can leverage these insights by promoting interprofessional education and training, developing standardized processes for care coordination, incentivizing collaborative initiatives, and ensuring leadership commitment to patient safety. Investment in technology and infrastructure, such as interoperable electronic health records and secure communication platforms, can further enhance collaboration, improve patient outcomes, and strengthen a culture of safety and continuous learning. These recommendations provide practical guidance for advancing collaborative patient safety initiatives while addressing systemic barriers and promoting sustainable improvements in healthcare delivery.

### Limitations

This study acknowledges several limitations that could impact the generalizability and scope of its findings. The relatively small sample size of 20 participants may limit the applicability of the results to broader healthcare contexts. Additionally, the study’s participant pool was influenced by geographical constraints and participant availability, potentially limiting the diversity of perspectives captured. The researchers also acknowledge the potential for researcher bias to influence data collection, analysis, and interpretation, despite their efforts to mitigate this through transparency and reflexivity. These limitations highlight the need for future research with larger, more diverse samples, wider geographical reach, and robust strategies to address researcher bias. This would contribute to a more comprehensive understanding of collaborative patient safety practices and their impact across various healthcare settings.

## Conclusion

The findings show that interprofessional collaboration is critical for advancing patient safety, and strong leadership, organizational support, and a safety culture are essential for sustaining effective teamwork. Despite structural and cultural barriers, strategies such as interprofessional education, transparency, patient engagement, and leveraging technology can reduce obstacles and foster collaboration.

### Importance of interprofessional collaboration

Effective communication, shared decision-making, and mutual respect among team members prevent medical errors, improve care coordination, and strengthen patient outcomes.

### Role of leadership and organizational support

Committed leadership and clear accountability structures drive successful collaborative initiatives, while resource provision and fostering a safety-oriented culture are necessary for lasting change.

### Barriers to collaboration and solutions

Barriers—including hierarchy, siloed communication, turf battles, time pressures, and limited resources—impede teamwork. Cultural change, team-based training, trust-building, and system-level interventions are recommended solutions.

### Patient engagement and safety culture

Involving patients as partners, enhancing transparency, and ensuring accountability create a strong safety culture wherein all stakeholders feel empowered to participate in safety efforts.

### Best practices and lessons learned

Interdisciplinary training, simulation exercises, technology tools (like electronic health records), continuous quality monitoring, and regular feedback loops improve collaboration and care quality.

In conclusion, strengthening interprofessional collaboration and a robust safety culture—with leadership backing and genuine patient engagement—lays the foundation for improved patient safety and care quality.

## Supporting information

S1 File Semi-structured interview. (DOCX)

## References

[1] Almalki MAA, Alanazy AAS, Alenzi SMJ, Alharbi ZDM, Alanazi RG, Alhareth AMDA, et al. Quality assurance and safety culture in a healthcare organization. IJMSCRS. 2023;03(10). doi: 10.47191/ijmscrs/v3-i10-17

[2] Shenoy A. Patient safety from the perspective of quality management frameworks: a review. Patient Saf Surg. 2021;15(1):12. doi: 10.1186/s13037-021-00286-6 33752725 PMC7983197

[3] Sreeramoju PV, Palmore TN, Lee GM, Edmond MB, Patterson JE, Sepkowitz KA, et al. Institutional quality and patient safety programs: an overview for the healthcare epidemiologist. Infect Control Hosp Epidemiol. 2021;42(1):6–17. doi: 10.1017/ice.2020.409 32883390 PMC8905713

[4] Elliott RA, Camacho E, Jankovic D, Sculpher MJ, Faria R. Economic analysis of the prevalence and clinical and economic burden of medication error in England. BMJ Qual Saf. 2021;30(2):96–105. doi: 10.1136/bmjqs-2019-010206 32527980

[5] World Health Organization. Global patient safety action plan 2021–2030: towards eliminating avoidable harm in health care. Geneva: World Health Organization; 2021. https://www.who.int/publications/i/item/9789240032705

[6] Leveson N, Samost A, Dekker S, Finkelstein S, Raman J. A systems approach to analyzing and preventing hospital adverse events. J Patient Saf. 2020;16(2):162–7. doi: 10.1097/PTS.0000000000000263 26756729

[7] Saporta A. Exploring healthcare professionals’ attitudes toward patient safety and behaviors that foster safe patient care. California State University, Bakersfield. 2024. Available from: https://scholarworks.calstate.edu/concern/theses/ff365c88r

[8] Moreira FTLDS, Callou RCM, Albuquerque GA, Oliveira RM. Effective communication strategies for managing disruptive behaviors and promoting patient safety. Rev Gaucha Enferm. 2019;40(spe):e20180308. doi: 10.1590/1983-1447.2019.20180308 31038600

[9] Wong BM, Baum KD, Headrick LA, Holmboe ES, Moss F, Ogrinc G, et al. Building the bridge to quality: an urgent call to integrate quality improvement and patient safety education with clinical care. Acad Med. 2020;95(1):59–68. doi: 10.1097/ACM.0000000000002937 31397709

[10] Steven A, Tella S, Turunen H, Flores Vizcaya-Moreno M, Pérez-Cañaveras RM, Porras J, et al. Shared learning from national to international contexts: a research and innovation collaboration to enhance education for patient safety. J Res Nurs. 2019;24(3–4):149–64. doi: 10.1177/1744987118824628 34394520 PMC7932281

[11] Lalani M, Wytrykowski S, Hogan H. Approaches to improving patient safety in integrated care: a scoping review. BMJ Open. 2023;13(4):e067441. doi: 10.1136/bmjopen-2022-067441 37015799 PMC10083780

[12] Wyss-Hänecke R, Lauener SK, Sluka C, Deschodt M, Siqeca F, Schwendimann R. Implementation fidelity of a multifactorial in-hospital fall prevention program and its association with unit systems factors: a single center, cross-sectional study. BMC Health Serv Res. 2023;23(1):158. doi: 10.1186/s12913-023-09157-5 36793084 PMC9930071

[13] Ricciardi W, Cascini F. Guidelines and safety practices for improving patient safety. J Transl Sci. 2021;3:3–18. doi: 10.29328/journal.jts.1001025 36315770

[14] Domer G, Gallagher TM, Shahabzada S, Sotherland J, Paul EN, Kumar K-N, et al. Patient safety: preventing patient harm and building capacity for patient safety. In: Domer G, editor. Contemporary Topics in Patient Safety-Volume 1. IntechOpen; 2021. pp. 1–22. doi: 10.5772/intechopen.94777

[15] Pedersen KZ, Mesman J. A transactional approach to patient safety: understanding safe care as a collaborative accomplishment. J Interprof Care. 2021;35(4):503–13. doi: 10.1080/13561820.2021.1874317 33653224

[16] Wang Y, Kung L, Gupta S, Ozdemir S. Leveraging big data analytics to improve quality of care in healthcare organizations: a configurational perspective. J Bus Res. 2019;30(2):362–88. doi: 10.1016/j.jbusres.2018.07.020

[17] Tamers SL, Streit J, Pana-Cryan R, Ray T, Syron L, Flynn MA, et al. Envisioning the future of work to safeguard the safety, health, and well-being of the workforce: a perspective from the CDC’s National Institute for Occupational Safety and Health. Am J Ind Med. 2020;63(12):1065–84. doi: 10.1002/ajim.23134 32926431 PMC7737298

[18] Newman B, Joseph K, Chauhan A, Seale H, Li J, Manias E, et al. Do patient engagement interventions work for all patients? A systematic review and realist synthesis of interventions to enhance patient safety. Health Expect. 2021;24(6):1905–23. doi: 10.1111/hex.13343 34432339 PMC8628590

[19] Agbar F, Zhang S, Wu Y, Mustafa M. Effect of patient safety education interventions on patient safety culture of health care professionals: Systematic review and meta-analysis. Nurs Educ Perspect. 2023;67:103565. doi: 10.1097/01.NEP.0000000000001035 36731258

[20] Fink-Samnick E. Leveraging interprofessional team-based care toward case management excellence: part 1, history, fundamentals, evidence. Prof Case Manag. 2019;24(3):130–41. doi: 10.1097/NCM.0000000000000344 30946250

[21] Greilich PE, Kilcullen M, Paquette S, Lazzara EH, Scielzo S, Hernandez J, et al. Team FIRST framework: Identifying core teamwork competencies critical to interprofessional healthcare curricula. J Clin Transl Sci. 2023;7(1):e106. doi: 10.1017/cts.2023.27 37250989 PMC10225264

[22] Boamah SA. Emergence of informal clinical leadership as a catalyst for improving patient care quality and job satisfaction. J Nurs Adm. 2019;75(5):1000–9. doi: 10.1097/NNA.0000000000000842 30375015

[23] Shirey MR, White-Williams C, Hites LJ. Integration of authentic leadership lens for building high performing interprofessional collaborative practice teams. Nurs Adm Q. 2019;43(2):101–12. doi: 10.1097/NAQ.0000000000000332 30839447

[24] Zajac S, Woods A, Tannenbaum S, Salas E, Holladay CL. Overcoming challenges to teamwork in healthcare: a team effectiveness framework and evidence-based guidance. Front Psychol. 2021;6:606445. doi: 10.3389/fpsyg.2015.00644

[25] Amoo ME, Bringardner J, Chen J-Y, Coyle EJ, Finnegan J, Kim CJ, et al. Breaking down the silos: Innovations for multidisciplinary programs. ASEE Virtual Annual Conference Content Access. 2020.

[26] Chegini Z, Arab-Zozani M, Shariful Islam SM, Tobiano G, Abbasgholizadeh Rahimi S. Barriers and facilitators to patient engagement in patient safety from patients and healthcare professionals’ perspectives: A systematic review and meta-synthesis. Nurs Forum. 2021. 10.1111/nuf.12635 34339525

[27] Engle RL, Mohr DC, Holmes SK, Seibert MN, Afable M, Leyson J, et al. Evidence-based practice and patient-centered care: doing both well. J Nurs Care Qual. 2021;46(3):174–84. doi: 10.1097/NCQ.0000000000000553 PMC8162222 31233424

[28] MacGillivray TE. Advancing the culture of patient safety and quality improvement. J Med Diagn Case Rep. 2020;16(3):192. doi: 10.4172/2327-5162.1000192 PMC7587327 33133354

[29] Al-Anazi AMF, Alanazi ML, Alresheedi AFN, Al-Rashidi AMO, Al-Harbi FS, Al-Mutairi AMA. Healthcare quality improvement: strategies for improving the quality and safety of healthcare delivery in healthcare organizations. J Sci Fin Manag. 2022;9(4):152–4. doi: 10.29328/journal.jsfm

