# Supplementary material for: Exploring collaborative strategies to improve patient safety in healthcare organizations: A qualitative study
Source: PLoS One. 2026 Jan 13;21(1):e0341022. doi: 10.1371/journal.pone.0341022 (PMC12798996; doi:10.1371/journal.pone.0341022)
Supplement: S1 File — (DOCX) [file pone.0341022.s001.docx]

Format of Semi-Structured Interview:

Introduction:

- Brief introduction of the researcher and the purpose of the interview
- Explaining the format of the interview (semi-structured) and assuring the interviewee of its confidentiality

Main Questions:

1. Can you share your experiences and perspectives on collaborative patient safety initiatives within healthcare settings?
2. What do you consider to be the essential components of successful interprofessional collaboration in promoting patient safety?
3. How important do you think leadership and organizational support are in driving collaborative patient safety efforts?
4. What barriers or challenges have you encountered in fostering effective teamwork and collaboration in healthcare?
5. How do you view the role of patient engagement and safety culture in enhancing patient safety outcomes?
6. Can you provide examples of best practices or successful strategies you have observed in collaborative patient safety initiatives?
7. How do you believe technology can support collaborative patient safety efforts in healthcare settings?
8. In your opinion, what are the key factors that contribute to a culture of continuous learning and improvement in patient safety practices?

Follow-up Questions:

- Can you elaborate on a specific experience or project related to collaborative patient safety that you found particularly impactful?
- How do you think healthcare organizations can overcome hierarchical structures and communication breakdowns to enhance collaboration?
- What recommendations would you give to healthcare leaders and policymakers to promote a culture of collaboration and patient safety within their institutions?
- How do you envision the future of collaborative patient safety initiatives evolving in healthcare?

Closing:

- Thanking the participant for their information and time
- Providing the participant with an opportunity to ask any questions or provide additional comments
- Reiterating confidentiality and thanking them for their participation
